# Cross-Sectional Association Between Employment Status and Self-Rated Health Among Middle-Aged Japanese Women: The Influence of Socioeconomic Conditions and Work-Life Conflict

**DOI:** 10.2188/jea.JE20190005

**Published:** 2020-09-05

**Authors:** Kaori Honjo, Hiroyasu Iso, Ai Ikeda, Kazumasa Yamagishi, Isao Saito, Tadahiro Kato, Nobufumi Yasuda, Kiyoshi Aoyagi, Kazuhiko Arima, Kiyomi Sakata, Kozo Tanno, Manami Inoue, Motoki Iwasaki, Taichi Shimazu, Atsushi Goto, Taiki Yamaji, Norie Sawada, Shoichiro Tsugane

**Affiliations:** 1 Social and Behavioral Sciences, Osaka Medical College, Faculty of Medicine, Osaka, Japan; 2 Public Health, Department of Social Medicine, Graduate School of Medicine, Osaka University, Osaka, Japan; 3 Department of Public Health, Graduate School of Medicine, Juntendo University, Tokyo, Japan; 4 Department of Public Health Medicine, Faculty of Medicine, University of Tsukuba, Ibaraki, Japan; 5 Department of Public Health and Epidemiology, Faculty of Medicine, Oita University, Oita, Japan; 6 Center for Education and Educational Research, Faculty of Education, Ehime University, Ehime, Japan; 7 Department of Public Health, Kochi Medical School, Kochi, Japan; 8 Department of Public Health, Nagasaki University, Graduate School of Biomedical Sciences, Nagasaki, Japan; 9 Department of Hygiene and Preventive Medicine, Iwate Medical University, Iwate, Japan; 10 Epidemiology and Prevention Group, Center for Public Health Sciences, National Cancer Center, Tokyo, Japan

**Keywords:** employment status, self-rated health, work-family conflict, Japan, women

## Abstract

**Background:**

Few studies examining the impact for women of employment status on health have considered domestic duties and responsibilities as well as household socioeconomic conditions. Moreover, to our knowledge, no studies have explored the influence of work-family conflict on the association between employment status and health. This research aimed to investigate the cross-sectional associations between employment status (regular employee, non-regular employee, or self-employed) with self-rated health among Japanese middle-aged working women.

**Methods:**

Self-report data were obtained from 21,450 working women aged 40–59 years enrolled in the Japan Public Health Center-based Prospective Study for the Next Generation (JPHC-NEXT Study) in 2011–2016. Multivariate odds ratios (ORs) and 95% confidence intervals (CIs) were calculated for poor self-rated health (‘poor’ or ‘not very good’) by employment status. Sub-group analyses by household income and marital status, as well as mediation analysis for work-family conflict, were also conducted.

**Results:**

Adjusted ORs for the poor self-rated health of non-regular employees and self-employed workers were 0.90 (95% CI, 0.83–0.98) and 0.84 (95% CI, 0.75–0.94), respectively, compared with regular employees. The identified association of non-regular employment was explained by work-family conflict. Subgroup analysis indicated no statistically significant modifying effects by household income and marital status.

**Conclusion:**

Among middle-aged working Japanese women, employment status was associated with self-rated health; non-regular employees and self-employed workers were less likely to report poor self-rated health, compared with regular employees. Lowered OR of poor self-rated health among non-regular employees may be explained by their reduced work-family conflict.

## INTRODUCTION

Employment status (ie, full-time, part-time, dispatch, contract, non-regular, or self-employed) may influence health.^1^^–^^4^ Precarious employment has been associated empirically with deteriorated health outcomes, using various measures of mortality,^2^^,^^3^^,^^5^^,^^6^ mental health,^7^^–^^9^ and health behaviors.^10^^,^^11^ However, inconsistent results have also been reported, particularly for self-rated health; non-regular workers have evidenced poorer self-rated health compared with regular workers,^12^^,^^13^ while some studies showed no significant differences^14^ or even a reverse association.^15^^–^^17^

One of the possible reasons for the inconsistent results could be underlying differences in participants’ reasons for being temporary workers.^6^ For example, some voluntarily choose temporary employment for domestic reasons, such as maintaining a work-family balance, while others are unable to find regular employment and are forced to accept temporary work. Further, the voluntariness of a job choice for non-regular employees may differ based on household socioeconomic conditions and may be influenced by varying societal norms regarding gender roles.^18^

In Japan, the number and proportion of female non-regular workers (ie, temporal, contract, and part-time) is much larger than that of non-regular male workers. In 2018, 54% of female employees and 14% of male employees aged 24–65 years old were non-regular workers.^19^ The gender role norms (ie, men work outside the home, women stay at home and take care of children and the elderly) that exist in Japanese society could be one of the reasons for this significant gender difference. Women’s participation in Japan’s workforce by age group shows a bimodal pattern. By contrast, similar to Japan’s male workforce, many Western societies display a convex shape.^20^ This bimodal pattern suggests that women tend to take a career break during their 20s and 30s, for such reasons as child rearing, and return to the workforce in their 40s.^21^^,^^22^ Part-time work is often the only choice for women who reenter the workforce^23^; it is an important path for women reentering the labor market.^24^ Consequently, a much higher proportion of women than men in Japan are non-regularly employed (mainly part-time).^19^

The proportion of female non-regular workers has increased along with the promotion of women’s participation in the workforce in the last few decades in Japan.^25^ However, these promotional efforts have not improved the imbalanced participation in household duties. That is, in most societies including Japan, women’s household responsibilities have persisted despite more women entering and playing a greater role in the workforce.^26^^–^^28^ Work-family conflict, generally defined as “a form of inter-role conflict in which the role pressures from the work and family domains are mutually incompatible in some respect,”^29^ has been associated with women’s self-rated health.^30^^–^^33^ An international comparison study showed that Japanese female workers had the highest work-family conflict and poorest self-rated mental and physical health among Japanese, Finnish, and English government workers.^34^ Other research, moreover, has shown that the working arrangement is associated with the prevalence of work-family conflict, with higher work-family conflict existing among full-time versus part-time workers.^33^^,^^35^ Women in Japan are often obligated to work part-time to mitigate their burden of managing both work and household demands and responsibilities because non-regular employment often offers more schedule flexibility compared with regular employment.^36^^,^^37^ Thus, we hypothesized that the association between employment status and self-rated health may be explained by the level of work-family conflict. Indeed, one of the main reasons middle-aged women in Japan choose non-regular employment and self-employment is time flexibility,^19^ which can reduce work-family conflict raised by multiple social roles for working women.^38^ To our knowledge, no studies have explored the effect of work-family conflicts on the association between employment status and health.

Available financial and material resources can affect the conditions relating to employment status. For example, some women work part-time to make a living, while others only need to boost the household budget; such differences produce heterogeneity amongst non-regular employees.^18^ Thus, household socioeconomic conditions may impact the association between employment status and health.^26^^–^^28^ However, few studies have included domestic duties and responsibilities and household socioeconomic conditions in examinations of the impact of employment status on health among women.

The current study thus aimed to investigate the associations of employment status (ie, regular, non-regular, or self-employed) with self-rated health among Japanese middle-aged working women. Our specific research questions were:

1) Is employment status associated with the probability of having poor self-rated health among Japanese working women?
2) Is the association between employment status and self-rated health explained by level of work-family conflict?
3) Are the associations between employment status and self-rated health noted above modified by socioeconomic conditions?

## MATERIAL AND METHODS

### Study cohort

Data in this study were derived from the Japan Public Health Center-based Prospective Study for the Next Generation (JPHC-NEXT Study). The JPHC-NEXT study was initiated in 2011 and the baseline survey was completed by December 31, 2016. In 2011–2016, we established a population-based cohort of 261,939 residents aged 40–74 years who registered an address in cities, towns, and villages (16 total) in seven prefectures throughout Japan. A self-administered questionnaire was distributed mostly by hand and partly by post to all participants; questions were asked about their lifestyles, personal medical histories, and socio-demographics. Incomplete answers were followed up by telephone interview. Among 261,939 residents, 115,385 agreed to participate in the JPHC-NEXT Study (response rate, 44.1%). All participants provided written informed consent. We excluded those with did not respond to the questionnaire. Our final cohort population was 114,157 (52,572 men and 61,585 women).^39^

The JPHC-NEXT Study was approved by the institutional review boards of the National Cancer Center and other participating institutions.

### Study population

We excluded women aged 60 years and older, women who were unemployed, and women for whom no information on occupation was available, to restrict our study population to working women 40–59 years old (*n* = 24,375). From these, we excluded 1,778 women with a history of cancer or cardiovascular disease and/or physical limitations; we excluded a further 1,147 women with undisclosed employment status, self-rated health, and/or work-family conflict scores. The final study population comprised 21,450 women.

### Measurements

#### Predictor variable

Our main predictor variable was employment status. Employment status was identified using self-report and categorized into three groups: 1) regular employee, 2) non-regular employee (ie, part-time, temporary, or contract worker), and 3) self-employee.

#### Outcome variable

We measured self-rated health as our outcome variable, using the single questionnaire item: “How would you describe your overall state of health?”. Participants chose one of five responses: poor, not very good, good, very good, and excellent. Following previous studies,^40^^,^^41^ we categorized the responses into two groups: 1) poor self-rated health (‘poor’ or ‘not very good’), and 2) not poor self-rated health (‘good,’ ‘very good,’ or ‘excellent health’).

#### Covariates

Age; highest education attainment level (junior high school, high school, junior college, college and higher, and other); occupation category (professional/managerial, clerical, or manual job); equivalent household income (quintile); marital status (married and non-married); history of hypertension, diabetes mellitus, hypercholesterolemia, and any other diseases (gout, asthma, chronic obstructive pulmonary disease, chronic bronchitis, chronic kidney failure, cataract, glaucoma, gastric polyp, colon polyp, gastric ulcer, duodenal ulcer, hepatic cirrhosis, hepatitis, gall stone, sleep apnea, or depression); and residential area were hypothesized as confounding factors. With regard to income, we obtained the data for annual household income using six categories. We calculated household equivalent income by dividing household income (ie, inserted median value of each category) by the square root of total household members.^42^ Equivalent household income was classified into quintiles; we also categorized equivalent household income into two groups by its median (High and Low) for sub-group analysis.

The data for work-family conflict was obtained through a self-reported questionnaire. Previous studies suggest that work-family conflict consists of two factors (work-to-family and family-to-work conflicts), which can be combined as a joint scale.^43^^,^^44^ The questionnaire for work-family conflict consisted of eight items (four items each for work-to-family conflict and family-to-work conflict; eTable 1), which were adapted from the United States National Study of Midlife Development^43^ and others.^33^^,^^34^ Each question had three response categories (0 = never, 1 = to some extent, and 2 = often). The responses to the eight items were summed up to yield a measure for work-family conflict scores ranging from 0 to 16. The internal consistency of work-family conflict in this study population was deemed acceptable (Cronbach’s alpha = 0.81). Further evidence on the reliability and validity of these scales have been provided by several other studies.^33^^,^^34^ The scores for work-family conflict were grouped into tertiles for the analysis in accordance with previous studies.^34^

### Statistical analysis

We calculated the number (%) or mean (standard deviation [SD]) of the study population’s characteristics. Using chi-square tests and analysis of variance, differences among employment statuses were calculated in terms of proportion and mean values for poor self-rated health, socioeconomic variables, marital status, medical history of hypertension, diabetes mellitus, or hypercholesterolemia, history of diseases, and work-family conflict scores.

Odds ratios (ORs) with 95% confidence intervals (CIs) of employment status for poor self-rated health were calculated using logistic regression analysis after adjusting for hypothesized confounding variables, such as age, education level, household equivalent income, occupation, marital status, medical history of hypertension, diabetes mellitus, hypercholesterolemia, or any other diseases, and residential area (model 1). To explore the mediating effect of work-family conflict on the association between employment status and self-rated health, we included work-family conflict scores in the model (model 2). Further, we conducted mediation analysis and estimated direct and indirect effects of employment status on self-rated health through work-family conflict. The natural direct effect (NDE), natural indirect effects (NIE), and total effects (TE) were estimated as ORs for poor self-rated health with respect to the mediator, conditioned on the measured covariates.^45^ We also estimated the percentage of the total associations between employment status and self-rated health that were mediated through work-family conflict using log ORs.^46^

Sub-group analyses by household equivalent income and marital status were also performed for self-rated health. We tested statistical interactions using cross-product terms for employment status and household equivalent income or marital status. All analyses were performed using SAS version 9.4 (SAS Institute Inc. Cary, NC, USA).

## RESULTS

Table 1 shows the number (%) and means (SD) of the study population’s characteristics. The average age was 50.3 (SD, 6.08) years; 17.7% of participants had poor self-rated health. Participants with regular employment, non-regular employment, and self-employment represented 40.6%, 43.2%, and 16.2% of the study population, respectively. The proportion of those who had attained a high school education or less was 55.2%, while those with undergraduate degrees or higher was 8.1%. Thirty percent of participants held professional/managerial jobs and 79.8% were married. The distributions of poor self-rated health differed by employment status: 18.9% for regular employees, 17.4% for non-regular employees, and 15.3% for self-employed. Compared with the other employment types, regular employees tended to be younger, managers/professionals, non-married, more educated, to have a higher household income, a medical history of diseases, and higher work-family conflict.

**Table 1.** Characteristics of study population according to employment status

					Employment type				
				
	ALL		Regular employee		Non-regular employee		Self-employee		*P*-value for difference
	21,450		8,708		9,272		3,470		
			
	*n*	%	*n*	%	*n*	%	*n*	%	
***n* (%)**									
Poor Self-rated Health									0.0001
Yes	3,789	17.7	1,643	18.9	1,614	17.4	532	15.3	

Education level									<.0001
Junior High School graduates	900	4.2	237	2.7	458	4.9	205	5.9	
High School graduates	10,938	51.0	3,801	43.7	5,301	57.2	1,836	52.9	
Junior college graduates	7,751	36.1	3,578	41.1	3,001	32.4	1,172	33.8	
University graduates and higher	1,739	8.1	1,041	12.0	458	26.3	240	6.9	
Others	87	0.4	34	0.4	39	44.8	14	16.1	
Missing	35	0.2	17	0.2	15	42.9	3	0.1	

Household equivalent income									<.0001
Lowest 0	3,505	16.3	978	11.2	2,054	22.2	473	13.6	
1	4,590	21.4	1,506	17.3	2,439	26.3	645	18.6	
2	3,278	15.3	1,390	16.0	1,376	14.8	512	14.8	
3	4,319	20.1	2,062	23.7	15,559	16.8	698	20.1	
Highest 4	4,460	20.8	2,226	25.6	1,320	14.2	914	26.3	
Missing	1,298	6.1	546	6.3	524	5.7	228	6.6	
Occupation									<.0001
Professionals & managers	6,421	29.9	3,873	44.5	2,004	21.6	544	15.7	
Clearical job	10,236	47.7	3,597	41.3	5,390	58.1	1,249	36.0	
Manual job	4,793	22.3	1,238	14.2	1,878	20.3	1,677	48.3	

Marital status									<.0001
Married	17,107	79.8	6,508	74.7	7,527	81.2	3,072	88.5	
Non-married	4,272	19.9	2,169	24.9	1,716	18.5	387	11.2	
Missing	71	0.3	31	0.4	29	0.3	11	0.3	

Hypertension									0.05
Yes	1,881	8.8	763	8.8	779	8.4	339	9.8	
Diabetes									0.0462
Yes	448	2.1	167	1.9	190	2.1	91	2.6	
Hypercholesterolemia									0.0433
Yes	2,089	9.7	895	10.3	851	9.2	343	9.9	
Medical history of diseases^a^									<.0001
Yes	4,631	21.6	2,017	23.2	1,876	20.2	738	21.3	
Work-Family Conflict score									<.0001
0 (lowest)	5,890	27.5	1,808	20.8	2,953	31.9	1,129	32.5	
1	9,212	43.0	3,765	43.2	4,088	44.1	1,359	39.2	
2(highest)	6,348	29.6	3,135	36.0	2,231	24.1	982	28.3	
**Mean (SD)**									
Age	50	6.1	50	6.0	50	6.1	50	6.1	<.0001

SD, standard deviation.

^a^Diseases: heart disease, gout, asthma, COPD, chronic bronchitis, chronic kidney failure, cataracts, glaucoma, gastric polyp, colon polyp, gastric ulcer, duodenal ulcer, hepatitis/hepatic cirrhosis, gallstone, sleep apnea, or depression.

Table 2 presents crude and adjusted ORs (95% CI) of employment status for self-rated poor health. The multivariate ORs for poor self-rated health of non-regular employees and the self-employed, compared with that of regular employees, were 0.90 (95% CI, 0.83–0.98) and 0.84 (95% CI, 0.75–0.94), respectively (model 1). Moreover, the lower OR for poor self-rated health was attenuated after adjusting for work-family conflict and we observed no significant differences in odds between regular employees and non-regular employees (OR 1.00; 95% CI, 0.92–1.09) (model 2). However, while the lower OR was attenuated, a statistically significant association with poor self-rated health was identified after adjusting for work-family conflict among the self-employed (OR 0.87; 95% CI, 0.78–0.98).

**Table 2.** Adjusted ORs of employment type for poor self-rated health among Japanese middle-aged women.

	*n*	*n* of subjects with poor self-rated health	Crude model	Model 1^a^	Model 2^b^
		
			OR (95% CI)	OR (95% CI)	OR (95% CI)
**Employment type**					
Regular employee	8,708	1,643	1.00	1.00	1.00
Non-regular employee	9,272	1,614	0.91 (0.84, 0.98)	0.90 (0.83, 0.98)	1.00 (0.92, 1.09)
Self-employed	3,470	532	0.78 (0.70, 0.87)	0.84 (0.75, 0.94)	0.87 (0.78, 0.98)

**Work-Family conflict (0–2)**					
0	5,890	645			1.00
1	9,212	1,432			1.50 (1.36, 1.66)
2	6,348	1,712			3.08 (2.78, 3.41)

CI, confidence interval; OR, odds ratio.

^a^Model 1: adjusted by education level, equivalent household income, occupation category, age group, marital status, hypertension, diabetes, and hypercholesterolemia, history of diseases (heart disease, gout, asthma, COPD, chronic bronchitis, chronic kidney failure, cataracts, glaucoma, gastric polyp, colon polyp, gastric ulcer, duodenal ulcer, hepatitis/hepatic cirrhosis, gallstone, sleep apnea, or depression), and residential area.

^b^Model 2: Model 1+ Work-Family conflict

The results of mediation analysis by work-family conflict scores showed that the association between non-regular employment and self-rated health was largely mediated through work-family conflict (the proportion mediated through work-family conflict was 100%), and no direct effect of non-regular employment on self-rated health was identified (Table 3). Part (31%) of the total effect of self-employment on self-rated health was mediated by work-family conflict, but it was mainly a direct effect.

**Table 3.** Mediation analysis between employment type and poor self-rated health through work-family conflict among Japanese middle-aged women

**ALL *n* = 21,450**	Non-regular employee			Self-employed		
	
	OR	95% CI	%mediated	OR	95% CI	%mediated
Total effect OR^a^ (95% CI)	0.90	(0.82, 0.97)		0.82	(0.73, 0.92)	
Direct effect OR^a^ (95% CI)	1.00	(0.92, 1.09)	0%	0.88	(0.78, 0.99)	65%
Indirect effect OR^a^ (95% CI)	0.89	(0.88, 0.90)	100%	0.94	(0.92, 0.95)	31%

CI, confidence interval; OR, odds ratio.

^a^Adjusted by education level, equivalent household income, occupation category, age group, marital status, hypertension, diabetes, and hypercholesterolemia, history of diseases (heart disease, gout, asthma, COPD, chronic bronchitis, chronic kidney failure, cataracts, glaucoma, gastric polyp, colon polyp, gastric ulcer, duodenal ulcer, hepatitis/hepatic cirrhosis, gallstone, sleep apnea, or depression), and residential area.

Table 4 presents the results of sub-group analysis by household income and marital status. The adjusted ORs of non-regular employees and self-employees, compared with regular employees, were 0.83 (95% CI, 0.74–0.94) and 0.78 (95% CI, 0.66–0.92), respectively, in the higher household income group and 0.88 (95% CI, 0.80–0.96) and 0.81 (95% CI, 0.72–0.92), respectively, in the married group, while no statistically significant associations were identified in their counterpart groups. The association between employment status and self-rated health was more evident in the high household income group and in the married group, although they were not statistically significant.

**Table 4.** Adjusted^a^ ORs of employment type for poor self-rated health among Japanese middle-aged women, stratified by household income level/marital status

**Employment status**	*n*	*n* of subjects with poor self-rated health	OR^a^ (95% CI)	
**Household equivalent income**				
**High**				
Regular employee	5,121	954	1.00	
Non-regular employee	3,724	590	0.83 (0.74, 0.94)	0.62
Self-employed	1,908	269	0.78 (0.66, 0.92)	0.57
**Low**				
Regular employee	3,041	581	1.00	
Non-regular employee	5,024	923	0.96 (0.85, 1.08)	
Self-employed	1,334	223	0.90 (0.75, 1.07)	

**Marital status**				
**Married**				
Regular employee	6,508	1,186	1.00	
Non-regular employee	7,527	1,246	0.88 (0.80, 0.96)	0.19
Self-employed	3,072	454	0.81 (0.72, 0.92)	0.34
**Non-married**				
Regular employee	2,169	450	1.00	
Non-regular employee	1,716	362	0.96 (0.80, 1.13)	
Self-employed	387	75	0.91 (0.69, 1.21)	

CI, confidence interval; OR, odds ratio.

^a^Adjusted by education level, equivalent household income, occupation category, age group, marital status, hypertension, diabetes, and hypercholesterolemia, history of diseases (heart disease, gout, asthma, COPD, chronic bronchitis, chronic kidney failure, cataracts, glaucoma, gastric polyp, colon polyp, gastric ulcer, duodenal ulcer, hepatitis/hepatic cirrhosis, gallstone, sleep apnea, or depression), and residential area.

## DISCUSSION

In this study of middle-aged Japanese working women, non-regular employees and the self-employed were more likely to have poorer self-rated health compared with regular employees. Furthermore, the apparently lower probability of having poor self-rated health among non-regular employees was explained by their diminished level of work-family conflicts. The association between employment status and self-rated health differed by household income level and marital status, although these interactions were not statistically significant.

Our results are in line with other research findings^15^^–^^17^; for example, cross-sectional studies among public sector employees in Finland showed lower ORs for fixed-term female employees’ poor self-rated health, compared with female permanent employees (OR 0.70; 95% CI, 0.60–0.82).^17^ Although only a limited number of similar studies have been conducted in Japan, our results were consistent with those of the Comprehensive National Survey among 18–64-year-old Japanese female employees. They reported that, compared with permanent employment, unstable employment with short working hours was associated with a smaller proportion of women who rated their health as poor.^47^

One of the reasons for poorer self-rated health among regular employees compared with non-regular employees could be the difficulties that women in Japan face in reconciling family life and career. Female workers in Japan who play a significant part of the total workforce are also more likely to be influenced by strong societal gender norms to fulfill household duties.^24^ Thus, working as regular employees often leads to severe physical and psychosocial hardship for women.^38^ Women in Japan who were regular employees were more likely to report job pressures and inflexible work schedules and to experience more strain related to work and family than their non-regularly employed counterparts.^48^ Moreover, Japanese female workers were reported to have the highest work-life conflict and poorest self-rated mental health among Japanese, Finnish, and English government workers.^34^ Thus, female workers in Japan may voluntarily choose to work part-time to achieve a balance between their work and family lives.

Our results (ie, significantly lower work-family conflict among non-regular employees versus regular employees, effects of non-regular employment on poor self-rated health was mostly explained by work-family conflict) suggest that non-regular employees may have achieved better self-rated health than regular employees by buffering their work-family conflict. The possible benefits of non-regular employment, such as schedule flexibility and/or fewer duties and responsibilities at work, can reduce their work-family conflict, which may contribute to their reduced probability of having poor self-rated health. Our findings also suggest that Japanese female workers may find relief from difficult life situations caused by juggling work and family lives by taking non-regular jobs or being self-employed, which may also be beneficial for their self-rated health.

Our results were, however, inconsistent with previous work that showed a higher mortality risk for non-regular female employees than for regular employees in Japan.^5^ While we do not have a clear explanation for this inconsistency, we speculate that one possible reason could be differences between two outcomes in the mechanisms of the health impact of employment status. That is, self-rated health reflects current life conditions on a short-term basis, while mortality may reflect chronic and continued effects relating to employment such as job insecurity, financial insecurity, and social welfare. These chronic health effects may be particularly strong in Japan, where there is little employment flexibility and the gap between regular and non-regular employees is relatively large.^24^ In other words, being a non-regular employee may alleviate the psychosocial and physical burdens temporarily; however, it may lead to long-term health deterioration through chronic psychosocial and socioeconomic impacts. In addition, we identified that work-family conflict explained most of the association between employment status and the risk of having poor self-rated health in this study. Controlling for the influence of work-family conflict, we identified no significant differences in probabilities of having poor self-rated health by employment status. In other words, most of the differences observed in self-rated health by employment status may be attributed to differences in psychological well-being. We do not deny the effect of psychological health on mortality; however, we speculate that the lower risk of self-rated health among non-regular employees, mostly explained by psychological health, may not directly reflect the results for mortality.

Self-employed workers also showed better self-rated health compared with regular employees. Job autonomy, job control, and/or control of working hours are hypothesized as possible benefits of being self-employed.^49^ In contrast to non-regular employees, work-family conflict did not appear to explain the identified association between self-employment and poor self-rated health. Work-family conflict is constructed to show the tension between two mutually incompatible domains, work and family.^29^ The work and family lives of self-employed workers may not present as much conflict as those of the other types of employees because the two domains (work and family) are physically closer and there is greater job autonomy. Further research is needed to understand the mechanisms of the association between being self-employment and self-rated health.

Although we did not identify statistically significant interaction of socioeconomic conditions and employment status on self-rated health, the benefits of being a non-regular employee or being self-employed was greater among women with higher socioeconomic status (ie, high household income and married). Married women and women with a higher socioeconomic background are, arguably, more likely to be working to add extra income to that of the other breadwinner in the household. Thus, they can enjoy the flexibility of non-regular work, and, importantly, take advantage of it by reducing work-family conflict. By contrast, unmarried women and women with in lower socioeconomic conditions are more likely to be making their own living, which could make them vulnerable to the disadvantages of non-regular employment, such as low payment and poor social security.^24^

This study is one of the few to observe the association between employment status and self-rated health in Japan and to explore the associations that are related to work-life conflict. However, there are several limitations. First, given the cross-sectional nature of the study, we cannot claim causative links; in particular, we cannot exclude the possibility of reverse causation. To reduce this possibility, we excluded women with medical histories of major diseases and statistically controlled the medical history of diseases. We also conducted sensitivity analysis by further excluding women with medical histories of diseases, which did not change our conclusions (OR 0.88; 95% CI, 0.80–0.97 for non-regular employee and OR 0.80; 95% CI, 0.70–0.92 for self-employees). Further research is required to establish causative links. Second, our results may have been affected by selection bias caused by non-participation or exclusion because of missing values in our main variables. For example, if non-participation occurred disproportionally in self-rated health conditions or in employment status, our conclusions could have been distorted. We do not have specific information about the direction of bias. However, excluded women with missing values on self-rated health, employment status, and work-family conflict were likely to have lower socioeconomic status, which implies that our results may be underestimated. Third, our study population may not be nationally representative; in particular, we did not include metropolitan areas. In addition, our study population was limited to women aged in their 40s and 50s. They may be widely different from younger generation in terms of their life situations, life-styles, domestic duties and responsibilities, and work-life balance. Thus, the generalizability of our results to other populations requires caution. Finally, measurement errors in our variables, including our outcome and predictor and unmeasured (confounding) variables, may have resulted in residual confounding.

### Conclusions

Employment status is associated with probabilities of poor self-rated health among middle-aged working Japanese women; non-regular employees and self-employed workers were less likely to report poor self-rated health compared with regular employees. Additionally, most of the associations with the poor self-rated health of non-regular employment could be explained by the level of work-family conflict. We did not identify significant influences of household income and marital status on the association between employment status and self-rated health.

## APPENDIX A. SUPPLEMENTARY DATA

eTable 1. Work-Family Conflict score

eTable 2. Adjusted ORs of employment type for poor self-rated health among Japanese middle-aged women
