# Supplementary material for: Cross-Sectional Association Between Employment Status and Self-Rated Health Among Middle-Aged Japanese Women: The Influence of Socioeconomic Conditions and Work-Life Conflict
Source: J Epidemiol. 2020 Sep 5;30(9):396–403. doi: 10.2188/jea.JE20190005 (PMC7429146; doi:10.2188/jea.JE20190005)
Supplement: Supplementary file 1 [file je-30-396-s001.pdf]

eTable1. Work-Family Conflict score

**Work-to-family conflict**	
(i)	'Your job reduces the amount of time you can spend with the family'
(ii)	'Problems at work make you irritable at home'
(iii)	'Your work involves a lot of travel away from home'
(iv)	'Your job takes so much energy you do not feel up to doing things that need attention at home'
**Family-to-work conflict**	
(v)	'Family matters reduce the time you can devote to your job'
(vi)	'Family worries about problems distract you from your work'
(vii)	'Family activities stop you getting the amount of sleep you need to do your job well'

eTable 2. Adjusted ORs of employment type for poor self-rated health among Japanese middle-aged women

	n	n of subjects with poor self-rated	Crude model OR (95% CI)	Model 1[a] OR (95% CI)	Model 2[b] OR (95% CI)
**Employment type**					
Regular employee	8,708	1,643	1.00	1.00	1.00
Non-regular employee	9,272	1,614	0.91 (0.84 , 0.98)	0.90 (0.83 , 0.98)	1.00 (0.92 , 1.09)
Self-employed	3,470	532	0.78 (0.70 , 0.87)	0.84 (0.75 , 0.94)	0.87 (0.78 , 0.98)
**Age category**					
40-44	4,845	831	1.00	1.00	1.00
45-49	4,701	890	1.17 (1.06 , 1.30)	1.19 (1.08 , 1.32)	1.19 (1.07 , 1.31)
50-54	5,285	1,035	1.22 (1.11 , 1.35)	1.19 (1.08 , 1.32)	1.22 (1.10 , 1.35)
55-59	5,611	1,035	0.95 (0.86 , 1.05)	0.84 (0.76 , 0.94)	0.90 (0.81 , 1.00)
**Education level**					
Junior High School graduates	900	177	1.00	1.00	1.00
High School graduates	10,938	2,002	0.92 (0.77 , 1.09)	0.91 (0.77 , 1.09)	0.89 (0.74 , 1.06)
Junior college graduates	7,751	1,335	0.85 (0.71 , 1.01)	0.82 (0.68 , 0.99)	0.76 (0.63 , 0.92)
University graduates and higher	1,739	242	0.66 (0.53 , 0.82)	0.61 (0.49 , 0.77)	0.53 (0.42 , 0.67)
Others	87	24	1.56 (0.95 , 2.56)	1.38 (0.83 , 2.30)	1.39 (0.83 , 2.33)
Missing	35	9	1.41 (0.65 , 3.07)	1.43 (0.65 , 3.13)	1.36 (0.61 , 3.01)
**Household equivalent income**					
Lowest 0	3,505	648	1.00	1.00	1.00
1	4,590	866	1.03 (0.92 , 1.15)	0.97 (0.86 , 1.09)	1.02 (0.91 , 1.15)
2	3,278	557	0.90 (0.80 , 1.02)	0.90 (0.79 , 1.02)	0.91 (0.80 , 1.04)
3	4,319	744	0.92 (0.82 , 1.03)	0.88 (0.78 , 1.00)	0.93 (0.82 , 1.05)
Highest 4	4,460	725	0.86 (0.76 , 0.96)	0.84 (0.74 , 0.95)	0.88 (0.78 , 1.00)
Missing	1,298	249	1.05 (0.89 , 1.23)	0.93 (0.79 , 1.11)	1.12 (0.94 , 1.33)
**Occupation**					
Professionals & managers	6,421	1,193	1.00	1.00	1.00
Clearical job	10,236	1,779	0.92 (0.85 , 1.00)	0.89 (0.82 , 0.98)	0.97 (0.88 , 1.06)
Manual job	4,793	817	0.90 (0.82 , 0.99)	0.88 (0.78 , 0.98)	0.97 (0.86 , 1.09)
**Marital status**					
Married	17,107	2,886	1.00	1.00	1.00
Non-married	4,272	887	1.29 (1.19 , 1.41)	1.24 (1.13 , 1.35)	1.30 (1.18 , 1.42)
Missing	71	16	1.43 (0.82 , 2.51)	1.37 (0.78 , 2.42)	1.26 (0.71 , 2.25)
**Hypertension**					
Yes	1,881	452	1.54 (1.38 , 1.72)	1.40 (1.24 , 1.57)	1.42 (1.26 , 1.60)
**Diabetes**					
Yes	448	131	1.96 (1.59 , 2.41)	1.60 (1.29 , 1.98)	1.52 (1.22 , 1.90)
**Hypercholesterolemia**					
Yes	2,089	485	1.47 (1.32 , 1.64)	1.34 (1.19 , 1.50)	1.34 (1.19 , 1.50)
**Medical history of diseases[c]**					
Yes	4,631	1,203	1.93 (1.79 , 2.09)	1.89 (1.75 , 2.05)	1.79 (1.65 , 1.94)
**Work-Family conflict (0-2)**					
0	5,890	645	1.00		1.00
1	9,212	1,432	1.50 (1.36 , 1.65)		1.50 (1.36 , 1.66)
2	6,348	1,712	3.00 (2.72 , 3.32)		3.08 (2.78 , 3.41)

[a]Model 1: Adjusted by the variables listed in the table with residential area.

[b]Model 2: Model1+Work-Family conflict.

[c] Diseases: heart disease, gout, asthma, COPD, chronic bronchitis, chronic kidney failure, cataracts, glaucoma, gastric polyp, colon polyp, gastric ulcer, duodenal ulcer, hepatitis/hepatic cirrhosis, gallstone, sleep apnea, or depression.

CI, confidence interval; OR, odds ratio.
